# Inner wall temperature distribution measurement of the ladle based on cavity effective emissivity correction

**DOI:** 10.1038/s41598-022-05533-z

**Published:** 2022-01-28

**Authors:** Jun Liu, Yan-hui Huang, Ying Ci, Jiang-xiong Fang, Feng Yang, Nobes David

**Affiliations:** 1 School of Geophysics and Measurement-Control Technology, East China University of Technology, Nanchang, 330013 Jiangxi China; 2 School of Information Science and Engineering, Northeastern University, Shenyang, 110819 Liaoning China; 3 School of Civil Engineering, East China University of Technology, Nanchang, 330013 Jiangxi China

**Keywords:** Electrical and electronic engineering, Mechanical engineering

## Abstract

Inner wall temperature of ladle is closely related to the quality of steelmaking and control of steel-making tapping temperature. This article adopts a rotating platform to drive an infrared temperature sensor and a laser sensor to scan the temperature field distribution of the ladle inner wall at the hot repair station, where the scanning laser sensor obtains coordinates of each measured point. Because of measuring errors of infrared thermal radiation caused by emissivity uncertainty of the ladle inner wall surface, this article proposes a method for temperature measurement based on Monte Carlo model for effective emissivity correction of each measured point. In the model, we consider the ladle and fire baffle as a cavity. By calculation of the model, the effect of distance from the fire baffle to the ladle and the material surface emissivity of the ladle inner wall on the effective emissivity of the cavity are obtained. After that, the effective emissivity of each measured point is determined. Then the scanning temperature of each measured point is corrected to real temperature. By field measuring test and verification contrast, the results show that: the maximum absolute error of the method in this article is 4.7 °C, the minimum error is 0.6 °C, and the average error is less than 2.8 °C. The method in this article achieves high measurement accuracy and contributes to the control of metallurgical process based on temperature information.

## Introduction

In continuous casting production, the ladle is the intermediate container that connects steelmaking and pouring. Temperature of the ladle is one of the important parameters affecting tapping temperature, quality and service life of the ladle refractory. If the temperature of the ladle is too low, inclusions in molten steel can not float up sufficiently, it will directly affect the quality of steel tapping and even cause production accidents. In contrast, if the temperature of the ladle is too high, this will shorten service life of the ladle refractory and increase the cost of steel-making, the cost per ton of steel will increase about by 1 yuan for every 1 °C increase in tapping temperature. In the field, because the ladle temperature is unknown, the tapping temperature is at least 20 °C higher than the ideal tapping temperature.

Therefore, it is necessary to acquire accurate temperature distribution of ladle inner wall, whose error is not greater than 5 °C, then the overall temperature of ladle can be obtained , and the purpose of ensuring steelmaking quality, safe production and cost saving can be under control.

At present, there are two kinds of direct measuring methods of ladle temperature, contact thermometry and non-contact thermometry. Contact thermometry is a direct and accurate measuring method which involves embedding or installing one or several thermocouples at set points. The thermocouples are in direct contact with the ladle wall or the inner lining of each layer. After sufficient heat exchange, the temperature distribution of the ladle (inner wall, inner lining, outer shell, etc.) can be obtained.

Phanomchoeng et al. embedded K-type and B-type thermocouples into each layer of the ladle to verify the online estimation of the ladle lining temperature^1^. Hoppmann et al. embedded two rows of 16 thermocouples in the working, permanent and insulation layer near the edge and bottom of the ladle. Besides, they installed thermocouples on the outer surface of the ladle, so as to measure the temperature distribution of the ladle^2^. Xiaodong et al. embedded 12 thermocouples symmetrically and evenly into each layer of ladle lining in furnace buildings^3^. Then variation of the ladle lining temperature in whole turnover process was obtained. Tian Jianguo embedded 8 thermocouples into the slag line above the ladle, the middle of the ladle, the bottom of the slag line and the interface of Working layer-permanent layer, permanent layer-ladle shell^4^. The relationship between thermal state of ladle and temperature drop of ladle was obtained by multiple regression method. Rieche et al. specially equipped a temperature gun with 6 thermocouples and insert it into the ladle to obtain the temperature distribution at different height of the ladle^5^.

The method of contact thermometry accurate^6–11^, however, the thermocouple is not only easily damaged during the turnover process because of the working layer of the ladle keeping peeling off, but also difficult to be repaired with a high temperature environment. In addition, this method is difficult to obtain the temperature field of ladle completely because of the quantitative limitation of measured points, it is usually used in the experiment or as the contrast verification to other methods.

Since the limitation of the contact thermometry, non-contact temperature thermometry is widely used in metallurgical field. The temperature distribution of the ladle is mainly measured by single-point infrared radiation pyrometer and thermal imager.

Jain, Dietmar, Kononov et al.^12–16^ adopted a single-point infrared radiation pyrometer to obtain the temperature of the ladle shell and verify the simulation results of the temperature field of the ladle insulation layer. In the single-point infrared radiation pyrometer temperature measurement, the emissivity of the measured object is set to a constant value^17–20^. However, the emissivity of the surface of the measured point is uncertain, which brings great error to the measurement.

Thermal imager is one of the popular tools for the temperature field measurement of the ladle^21–25^. Contrasted with single-point radiation thermometry, this method can obtain regional temperature information, but its principle is the same as that of infrared radiation thermometry, the temperature measuring error caused by the emissivity uncertainty of the measured object has not been solved substantially.

To obtain the ladle inner wall real temperature distribution, and solve emissivity uncertainty of the non-contact thermometry, this article studies a scanning measuring method of the ladle inner wall temperature distribution based on Monte Carlo model for correcting the effective emissivity of measured points on the ladle inner wall. The method adopts a rotating platform to drive an infrared temperature sensor and a laser sensor to scan the temperature distribution of the ladle inner wall at hot repair station, where the scanning laser sensor obtains coordinates of each temperature measured point. In order to solve the measuring errors of infrared radiation caused by emissivity uncertainty of the ladle inner wall surface, we establish Monte Carlo model for effective emissivity correction of each measured point. In the model, we consider the ladle and fire baffle as a cavity. By simulation calculation of the model, the effect of distance from the fire baffle to the ladle and the material surface emissivity of the ladle inner wall on the cavity effective emissivity are obtained. After that, the effective emissivity of each measured point is determined. Then the scanning temperature of each measured point is corrected to real temperature.

## Measurement principle

In order to obtain the temperature of the ladle before it is filled with molten steel, the hot repair station is selected as the measuring position where the ladle is ready to holding molten steel and its position is relatively fixed.

The temperature field measuring method in this article is composed of a rotating platform which is installed an infrared temperature sensor and a laser sensor to obtain the inner wall temperature field of the ladle inner wall, two positioning laser sensors, and a data calculation unit. After pouring, the ladle is carried by the crane back to the hot repair station. The infrared temperature sensor scans the inner wall of the ladle to obtain its temperature by the rotating platform. Although the position of the ladle is relatively fixed at the hot repair station, its absolute position cannot be guaranteed. Therefore, the positioning laser sensors aim to obtain accurate position of the ladle. The data calculation unit converts the scanning laser sensor data into the coordinates according to the positioning laser sensors data.

The principle and process of the measurement are:

1. Scanning measurement of the inner wall of the ladle. The rotating platform is an electric mechanism which can achieve specified horizontal and vertical azimuth angles according to command. The platform is also horizontally installed. In the measurement, the platform drives the infrared temperature sensor and the laser sensor to obtain the temperature measuring data and distance between the scanning laser sensor and the measured point on the inner wall of the ladle.
2. Ladle positioning. The two positioning lasers are horizontally installed. The plane of the two positioning laser sensors is defined as the positioning reference plane, each positioning laser sensor obtains the distance between a point on the bottom plane of the ladle and the positioning reference plane. According to two positioning distance, the offset position and angle of the bottom plane of the ladle relative to the positioning reference plane are obtained, and the positioning coordinates of the ladle shell are determined.
3. Coordinate mapping. In order to build the coordinates of the measured points on the inner wall of the ladle, the scanning distance data need to be converted into the ladle coordinate system. This article establishes the measurement coordinate system and the ladle coordinate system respectively, according to the installation position parameters and the positioning parameters which obtained by the positioning laser sensors. Then the scanning distance and azimuth angles of each measured point are mapped into the ladle coordinate system^17–20^.
4. Temperature measurement correction. We establish a Monte Carlo calculation model for the effective emissivity of the inner wall of the ladle. The scanning temperature of each measured point is corrected to the real temperature by the model. Finally, the temperature distribution of the ladle inner wall is obtained.

## Temperature field obtaining

### Temperature field scanning

**Figure 1:**
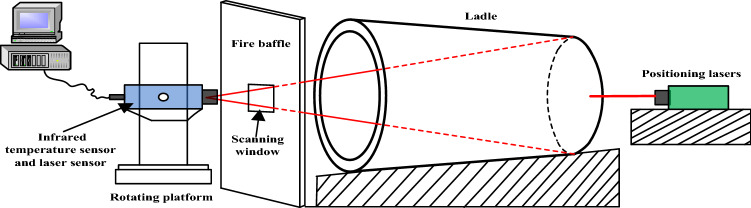
Schematic diagram of the temperature field scanning.

The schematic of the ladle inner wall temperature field scanning is shown in Fig. 1. The infrared temperature sensor and the laser sensor are installed on the rotating platform, which is arranged on the side of the ladle edge. When the ladle is located at the hot repair station, the platform drives the temperature sensor and the laser sensor to scan the ladle inner wall temperature and distance point by point from the scanning window of the fire baffle to obtain the ladle inner wall temperature distribution. The two positioning lasers are installed on the side of the ladle bottom to obtain the distance between the ladle bottom and the positioning lasers.

### Temperature field coordinate obtaining

The method in this article includes a positioning system and a scanning measuring system, both of which are installed in a fixed position, and the relative position of them is also fixed, so their spatial relationship can be determined offline. Because the ladle is located at the hot repair station each time with different offset position and angle. In this article, the distance between two points at the bottom plane of the ladle and positioning reference plane are obtained by positioning laser sensor #1 and positioning laser sensor #2, which are *L*1 and *L*2 respectively. Then the offset position of the bottom plane of the ladle to the positioning reference plane *L*_*d*_, and the offset angle *θ* are:

1 $$\left\{ {\begin{array}{*{20}l} {L_{d} = \frac{{L_{1} + L_{2} }}{2}} \\ {\theta = \arctan \frac{{\left| {L_{2} - L_{1} } \right|}}{d}} \\ \end{array} } \right.$$

where *d* is the distance between the center points of the two positioning laser sensors, which is a known value.

**Figure 2:**
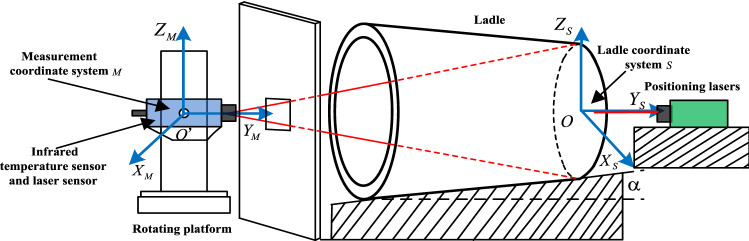
Coordinate systems of measurement.

In order to achieve the conversion of the scanning distance data of each measured point of the ladle inner wall into the ladle coordinate system, this article establishes two coordinate systems, namely measuring coordinate system *M* and ladle coordinate system *S* as shown in Fig. 2. In *M*, it considered the center point of the initial position of the scanning laser sensor *O*′ is to be the origin, direction on the horizontal plane parallel to the fire baffle plane to be *X*-axis, direction on the horizontal plane vertical to be *Y*-axis, and direction vertical to the horizontal plane to be *Z*-axis. The coordinate system *S* consider the center point of the bottom plane of the ladle shell *O* as the origin, direction perpendicular to the bottom plane as *Y*-axis, the vertical direction on the bottom plane as *Z*-axis, and direction on the bottom plane perpendicular to *Z*-axis as *X*-axis. In order to distinguish the two coordinate systems, the axes *X*, *Y*, *Z* of *M* are represented by *X*_*M*_, *Y*_*M*_, *Z*_*M*_, and coordinate in *M* is represented by (*x*_*m*_, *y*_*m*_, *z*_*m*_); similarly, the axes *X*, *Y*, *Z* of *S* are represented by *X*_*S*_, *Y*_*S*_, *Z*_*S*_, and coordinates in *S* is (*x*_*S*_, *y*_*S*_, *z*_*S*_).

At the hot repair station, due to the need of purging, the ladle is placed obliquely, whose bottom is higher than edge. The oblique angle between the ladle shell and the horizontal plane *α* is fixed. The displace between *O*′ and *O* along *X*_*S*_ and *Z*_*S*_ are *x*_0_ and *z*_0_ respectively, and the distance between *O*′ and the positioning reference plane is *L*. *x*_0_, *z*_0_, *L* can also be determined offline. So, if *L*_*d*_ and *θ* were determined, then the relationship between coordinates *M* and *S* is determined. Therefore, in order to obtain the coordinates of the measured points on the ladle inner wall, it is means to convert the scanning distance coordinates in *M* into the ones in *S*.

The displacement of *O*′ in *M* relative to *S* is ${{}^{S}P}_{M-org}={\left[{x}_{0},L-{L}_{d},{z}_{0}\right]}^{T}$, and the posture of *M* relative to *S* is ${}_{M}{}^{S}R$. The horizontal and vertical azimuth angles of the scanning laser sensor are $\beta$ and $\varphi$ which can be obtained in the measurement. The conversion relationship from *M* to *S* is:

2 $$p_{S} = {}_{M}^{S} R \cdot p_{M} + {}_{{}}^{s} p_{M - org}$$

where *p*_*s*_ are coordinates in *S*, and *p*_*M*_ are coordinates in *M*.

The coordinate system conversion formula is:

3 $$\begin{gathered} \left[ {\begin{array}{*{20}c} {x_{S} } \\ {y_{S} } \\ {z_{S} } \\ \end{array} } \right] = \left[ {\begin{array}{*{20}c} 1 & 0 & 0 \\ 0 & {\cos \left( { - \alpha } \right)} & { - \sin \left( { - \alpha } \right)} \\ 0 & {\sin \left( { - \alpha } \right)} & {\cos \left( { - \alpha } \right)} \\ \end{array} } \right]g\left[ {\begin{array}{*{20}c} {\cos \left( { - \theta } \right)} & { - \sin \left( { - \theta } \right)} & 0 \\ {\sin \left( { - \theta } \right)} & {\cos \left( { - \theta } \right)} & 0 \\ 0 & 0 & 1 \\ \end{array} } \right]g\left[ {\begin{array}{*{20}c} {\cos \beta } & { - \sin \beta } & 0 \\ {\sin \beta } & {\cos \beta } & 0 \\ 0 & 0 & 1 \\ \end{array} } \right]g\left[ {\begin{array}{*{20}c} 1 & 0 & 0 \\ 0 & {\cos \varphi } & { - \sin \varphi } \\ 0 & {\sin \varphi } & {\cos \varphi } \\ \end{array} } \right]g\left[ {\begin{array}{*{20}c} {x_{m} } \\ {y_{m} } \\ {z_{m} } \\ \end{array} } \right] + \left[ {\begin{array}{*{20}c} {x_{0} } \\ {\frac{{2L - L_{d} }}{2}} \\ {z_{0} } \\ \end{array} } \right] \hfill \\ = \left[ {\begin{array}{*{20}c} {x_{m} \cos \left( {\theta - \beta } \right) + y_{m} \sin \left( {\theta - \beta } \right)\cos \varphi - z_{m} \sin \left( {\theta - \beta } \right)\sin \varphi + x_{0} } \\ { - x_{m} \cos \alpha \sin \left( {\theta - \beta } \right) + y_{m} \left[ {\cos \alpha \cos \left( {\theta - \beta } \right)\cos \varphi + \sin \alpha \sin \varphi } \right] - z_{m} \left[ {\cos \alpha \cos \left( {\theta - \beta } \right)\sin \varphi + \sin \alpha \cos \varphi } \right] + \frac{{2L - L_{d} }}{2}} \\ {x_{m} \sin \alpha \sin \left( {\theta - \beta } \right) - y_{m} \left[ {\sin \alpha \cos \left( {\theta - \beta } \right)\cos \varphi - \cos \alpha \sin \varphi } \right] + z_{m} \left[ {\sin \alpha \cos \left( {\theta - \beta } \right)\sin \varphi - \cos \alpha \cos \varphi } \right] + z_{0} } \\ \end{array} } \right] \hfill \\ \end{gathered}$$

According to formula (3), the scanning distance data in measurement coordinate system can be converted into the ones in the ladle coordinate system, which ones are the coordinates of temperature measured points of the ladle inner wall.

## Monte Carlo effective emissivity calculation model

Temperature measurement of the ladle inner wall is achieved by obtaining the infrared radiation energy of the measured point by the infrared temperature sensor. Because the emissivity of the ladle inner wall surface is uncertainty, the emissivity is preset to be a constant value for the temperature sensor before the measurement. In this case, we obtain brightness temperature of the measured points rather than real temperature.

In this article, the inside structure of the ladle and fire baffle at the hot repair station can be considered as a combined cavity-like structure. Because of the advantages of Monte Carlo method for cavity and micro panel radiation characteristics^26–30^, this article establishes Monte Carlo calculation model for the effective emissivity of the ladle cavity based on reverse tracing of light to simulate the motion route of the infrared radiation light during the temperature measuring process, and then obtains the effective emissivity of each measured points on the ladle inner wall.

**Figure 3:**
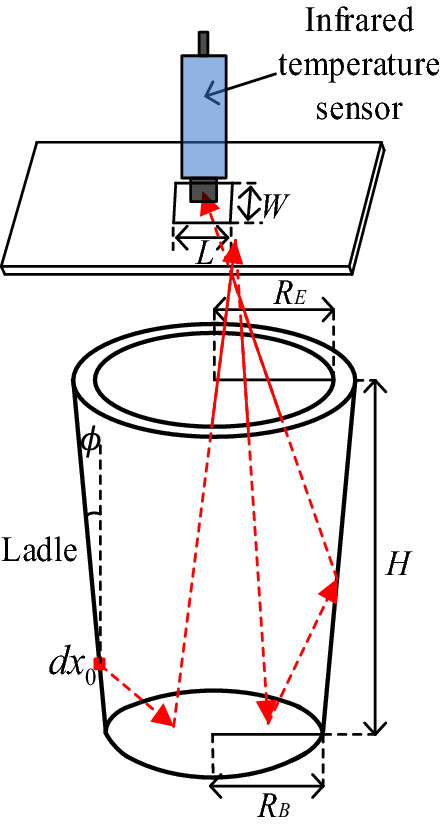
Motion route of the infrared radiation light in the cavity.

Motion route of the infrared radiation light in the cavity is shown in Fig. 3. Where ${R}_{E}$ is the radius of the ladle edge; ${R}_{B}$ is the radius of the bottom of ladle inner wall; $H$ is the height of the ladle inner wall; $\varphi$ is the bottom-edge angle of the ladle inner wall; $L$ is the length of the scanning window of the fire baffle, $W$ is the width of the window.

We assume that there are *N* beams of light incident to the micro panel *dx*_0_ (measured point) of the ladle inner wall from the measuring window, each beam of light carries the same radiant energy, and the effective absorption ratio ${\alpha }_{aD}^{0}$ of *dx*_0_ is defined as the energy ratio absorbed by the cavity of the total incident energy of *dx*_0_. While reference temperature of the cavity is *T*_0_, then:

4 $$\alpha_{aD}^{0} = \frac{{{{N}}_{\alpha } }}{{{N}}}$$

where ${N}_{\alpha }$ is the number of beams of light absorbed by the cavity. It then follows that:

5 $$\alpha_{aD}^{0} = \frac{{{N}_{\alpha } }}{N} = \frac{{{N}_{\alpha 0} }}{N} \cdot \frac{{{I}_{b} \left( {{T}_{x0} } \right)}}{{{I}_{b} \left( {{T}_{0} } \right)}} + \int \limits_{\sum \left( 1 \right)} \frac{{N_{\rho x1} }}{N} \cdot \frac{{{N}_{\alpha x1} }}{{{N}_{\rho x1} }} \cdot \frac{{{I}_{b} \left( {{T}_{x1} } \right)}}{{{I}_{b} \left( {{T}_{0} } \right)}} + \int \limits_{\sum \left( 1 \right)} \frac{{{N}_{\rho x1} }}{N} \cdot \int \limits_{\sum \left( 2 \right)} \frac{{{N}_{\rho x2} }}{{{N}_{\rho x1} }} \cdot \frac{{{N}_{\alpha x2} }}{{{N}_{\rho x2} }} \cdot \frac{{{I}_{b} \left( {{T}_{x2} } \right)}}{{{I}_{b} \left( {{T}_{0} } \right)}} + \cdots$$

where ${N}_{\alpha 0}$ is the number of beams of absorbed light at $dx_{0}$, ${N}_{\rho x1}$ is the number of beams of light which are reflected to micro panel $dx_{1}$ from $dx_{0}$, ${N}_{\alpha x1}$ is the number of beams of absorbed light at $dx_{1}$ which reflected from $dx_{0}$; ${T}_{xi}$ is the current temperature of micro panel $dx_{i}$; ${I}_{b} \left( {{T}_{xi} } \right)$ is the current radiant energy of $dx_{i}$; and $\sum \left( i \right)$ is the ratio of all beams of light energy absorbed after the $i$ th reflection.

On the premise of isothermal of the cavity, so that $\frac{{{I}_{b} \left( {{T}_{xi} } \right)}}{{{I}_{b} \left( {{T}_{0} } \right)}} = 1$, then Eq. (5) can be simplified to:

6 $${N}_{\alpha } = {N}_{\alpha 0} + {N}_{\alpha 1} + {N}_{\alpha 2} + \cdots + {N}_{\alpha i} + \cdots$$

In Eq. (6), ${N}_{\alpha i}$ means the beam of light is absorbed $i$ times.

When the cavity reaches thermal balance, The radiant energy emitted by $dx_{0}$ is equal to the absorbed energy of $dx_{0}$, therefore the emissivity of $dx_{0}$, $\varepsilon_{a0}^{D}$ equal to the absorption ratio $\alpha_{aD}^{0}$, so:

7 $$\varepsilon_{a0}^{D} = \alpha_{aD}^{0} = \frac{{{N}_{\alpha } }}{N} = 1 - \frac{{{N}_{\rho } }}{N}$$

where ${N}_{\rho }$ is the number of beams of escaped light from the cavity.

While the cavity is no-isothermal, the effective emissivity of the micro panel can be expressed as:

8 $$\varepsilon_{ai}^{D} \left( {\lambda ,T_{0} } \right) = \varepsilon_{ai}^{D} \left( {\lambda ,T_{0}^{^{\prime}} } \right)\frac{{E_{b} \left( {\lambda ,T_{0}^{^{\prime}} } \right)}}{{E_{b} \left( {\lambda ,T_{0} } \right)}}$$

where $\varepsilon_{ai}^{D} \left( {\lambda ,T_{0} } \right)$ is the effective emissivity of the micro panel at reference temperature $T_{0}$; $\varepsilon_{ai}^{D} \left( {\lambda ,T_{0}^{^{\prime}} } \right)$ is the effective emissivity of the micro panel at reference temperature $T_{0}^{^{\prime}}$; and $\frac{{E_{b} \left( {\lambda ,T_{0}^{^{\prime}} } \right)}}{{E_{b} \left( {\lambda ,T_{0} } \right)}}$ is the ratio of black body radiant energy at reference temperatures $T_{0}^{^{\prime}}$ and $T_{0}$.

Because the ladle inner wall is non-isothermal actually, a nonlinear no-isothermal coefficient $\Delta \zeta$ is adopted to correct the effective emissivity of each point along height direction on the ladle inner wall. $\varepsilon_{a0}^{D} \left( {\lambda ,T_{0}^{^{\prime}} } \right)$ can be obtained as:

9 $$\varepsilon_{a0}^{D} \left( {\lambda ,T_{0}^{^{\prime}} } \right) = \varepsilon_{a0}^{D} \left( {\lambda ,T_{0} } \right) \times \Delta \zeta$$

From the ladle edge to the bottom, $\Delta \zeta$ is as follow:

10 $$\Delta \zeta = \frac{1}{{1 + k \cdot \left( {e^{{ - \frac{{h_{i} }}{{H - h_{i} }}}} } \right)^{2} }}$$

where $H$ is the height of ladle inner wall; $h_{i}$ ($\in 0,H$) is height of micro panel $dx_{i}$; $k$ is verification coefficient to the ladle inner wall, which is obtained by the verification test of thermocouple.

## Analysis of the effect on the effective emissivity of the ladle

### The method of reverse tracing of light

**Figure 4:**
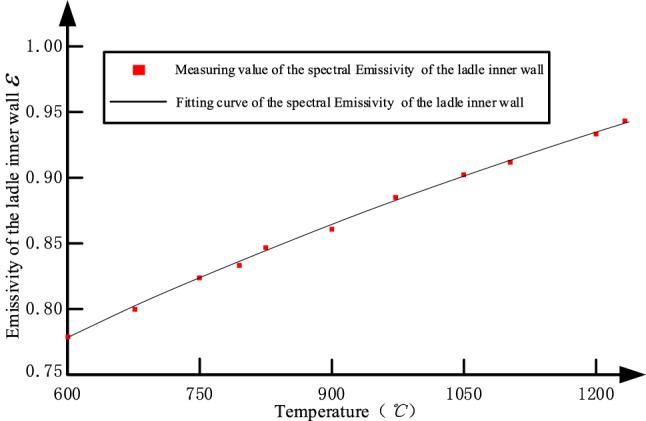
When *λ* = 1.05 μm, magnesia carbon material surface spectral emissivity with temperature variation.

In the field, the working lining of ladle inner wall is made of magnesia carbon brick. In the temperature measurement, we choose a stable measurement wavelength $\lambda$, which is 1.05 μm. While *λ* = 1.05 μm, the curve of magnesia carbon material surface spectral emissivity $\varepsilon$ with temperature variation is shown in Fig. 4. The curve is expressed as:

11 $$\varepsilon = 0.5491 + 0.0004399T + 0.0000001T^{2}$$

where $T$ is the real temperature of the observation point of the ladle inner wall.

Firstly, we set the material surface spectral emissivity to 1.0 and scan all the points on the ladle inner wall, then we obtain the scanning temperature and $\varepsilon$ of each point by formula (11). While in the filed test and verification, the temperature measuring error $\Delta T \le 50$ °C, the error of $\varepsilon$, $\Delta \varepsilon \in \;\left[ {0.01477,\;0.009745} \right]$ according to formula (11), $\Delta \varepsilon$ decreases with the increase of the temperature. And $\Delta \varepsilon$ can be ignored, it means that we assume the emissivity of the scanning temperature is consistent with the real temperature.

In this article, the method of reverse tracing of light includes following 6 steps:

1. Assume *N* beams of light incident to micro panel $dx_{0}$ on the ladle inner wall.
2. Set material surface emissivity of the intersection point $\varepsilon$ according to the scanning temperature and formula (11). Then adopt a random number $R_{e}$ to judge whether the light is absorbed or not. If $R_{e} \le \varepsilon$, the light is absorbed, add 1 to the number of absorbed beams ${N}_{\alpha }$, and continue to track the next beam of light.
3. If $R_{e} > \varepsilon$, the light is reflected. While, adopt another random number $R_{s}$ to judge whether the light is mirror reflected or diffuse reflected. If $R_{s} \le \rho_{s} /\rho$ ($\rho_{s} /\rho$ is the ratio specular reflection of the material), it is specular reflected, and determine its direction according to the law of geometric optics reflection. Otherwise, it is diffuse reflected, and two random numbers $R_{\theta }$ and $R_{\varphi }$ are adopted to determine its reflection direction.
4. Calculate the reflected intersection point on the inner wall. Repeat steps (2) to (3) until the beam is absorbed or escapes from the cavity.
5. Obtain the effective emissivity of this micro panel according to $\varepsilon_{a0}^{D} = \frac{{N_{a} }}{N}$.
6. If $\varepsilon < 1.0$, repeat steps (2) to (5), else perform step (7).
7. From the edge to the bottom, the measured points (which is consider as micro panel) are selected in sequence along the height direction of the inner wall. According to the nonlinear no-isothermal coefficient, $\varepsilon_{a0}^{D} \left( {\lambda ,T_{0}^{^{\prime}} } \right)$ with the variation of $\varepsilon$ is obtained point by point to complete the calculation of the effective emissivity of all points on the ladle inner wall.

### Calculation of the effective emissivity of the ladle inner wall

As the ladle inner wall is paved with refractory bricks, the emissivity of refractory bricks surface is effected by its composition, surface state, and temperature. The emissivity decreases with increasing of temperature, and the main effect factor is the surface state (the rougher the surface, the greater the emissivity). With rough surface, the refractory bricks surface emissivity is between 0.8 and 0.9 at 800–1200 °C. Based on this premise, the method is applied to the field of Hubei Xinyegang Steel Co., Ltd. According to the actual size of ladle in the field, parameters in Monte Carlo calculation model in this article are:

$R_{E} = 135 \;{\text{cm}}$, $R_{B} = 120\;{\text{cm}}$, $H = 450\;{\text{ cm}}$, $\varphi = {\text{arctan}}\left( {1/30} \right)$, $L = 37.5 \;{\text{cm}}$, $W = 37.5 \;{\text{cm}}$, $\varepsilon = 0.85$, $\rho_{s} /\rho = 0.03$, $N = 10^{8}$, the verification coefficient $k$ is 0.479.

As discussed above, the effective emissivity distribution of the whole ladle inner wall can be obtained. The main factors effect the effective emissivity $\varepsilon_{a0}^{D}$ of the points on the ladle inner wall are the distance between the fire baffle and the ladle $D_{C - M}$ and the material surface emissivity of the refractory bricks of inner wall $\varepsilon$.

Firstly, we analyze the effect of $D_{C - M}$ on $\varepsilon_{a0}^{D}$ of each point while $\varepsilon$ is 0.85, and then determine the value of $D_{C - M}$. Secondly, with determined $D_{C - M}$, we analyze the effect of $\varepsilon$ on $\varepsilon_{a0}^{D}$ of each point. And we select three representative points (the bottom point, the midpoint, and the edge point) along the height direction on the ladle inner wall as observation points.

**Figure 5:**
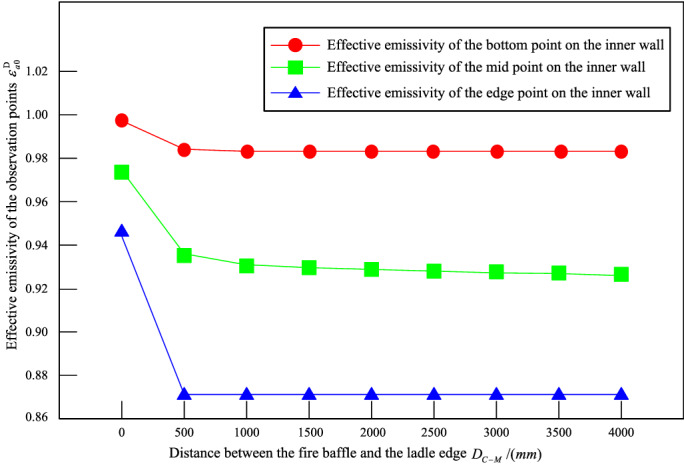
When $\varepsilon = 0.85$, the effect of $D_{C - M}$ on $\varepsilon_{a0}^{D}$ of each observation point.

The effect of $D_{C - M}$ on $\varepsilon_{a0}^{D}$ of each observation point, obtained by Monte Carlo calculation model is shown in Fig. 5.

It can be seen from Fig. 5 that $\varepsilon_{a0}^{D}$ decreases with the increase of $D_{C - M}$, and the larger the $D_{C - M}$ is, the smoother the decrease ratio of $\varepsilon_{a0}^{D}$ is. When $D_{C - M} \ge 1500\;{\text{mm}}$, $\varepsilon_{a0}^{D}$ tends to be stable ($\Delta \left| {\varepsilon_{a0}^{D} } \right| \le 0.005$). Therefore, according to the analysis results and the field condition, $D_{C - M}$ is determined as 1600 mm.

**Figure 6:**
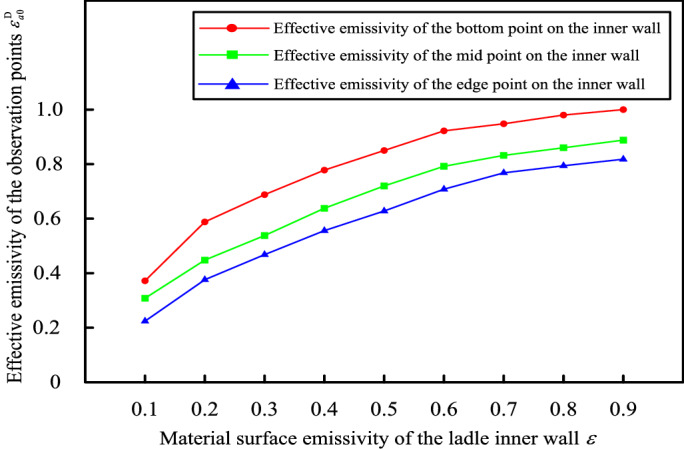
When $D_{C - M} = 1600\;{\text{mm}}$, the effect of $\varepsilon$ on $\varepsilon_{a0}^{D}$ of each observation point.

Under the conditions above, then the effect of $\varepsilon$ on $\varepsilon_{a0}^{D}$ analyzed by the model is shown in Fig. 6 .

It can be seen from Fig. 6 that the effective emissivity $\varepsilon_{a0}^{D}$ of each observation point on the ladle inner wall increases with the increase of the material surface emissivity $\varepsilon$, when $\varepsilon \ge 0.7$, the value of $\varepsilon_{a0}^{D}$ tends to be stable, especially when $\varepsilon \ge 0.8$, $\Delta \left| {\varepsilon_{a0}^{D} } \right| \le 0.02$.

## Test and verification

The ladle inner wall temperature field measurement method in this article is tested in the field of Hubei Xinyegang Steel Co., Ltd. the effective emissivity $\varepsilon_{a0}^{D}$ of each measured point are obtained by Monte Carlo calculation model.

**Figure 7:**
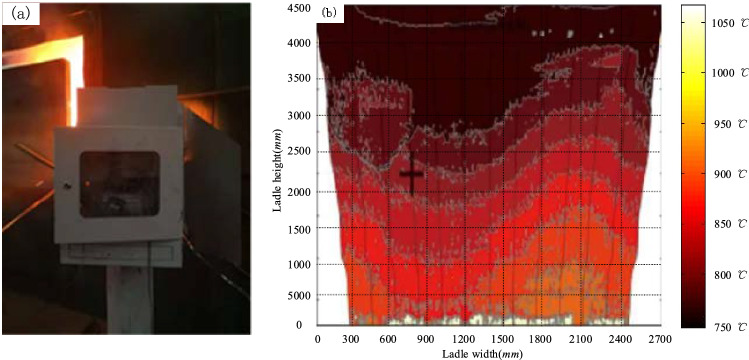
Field test (**a**) and the results of temperature measurement (**b**).

Finally, the real temperature $T$ of each measured point can be acquired according to the plank radiation law. The field test is shown in Fig. 7a, and the measuring result is shown in Fig. 7b.

**Figure 8:**
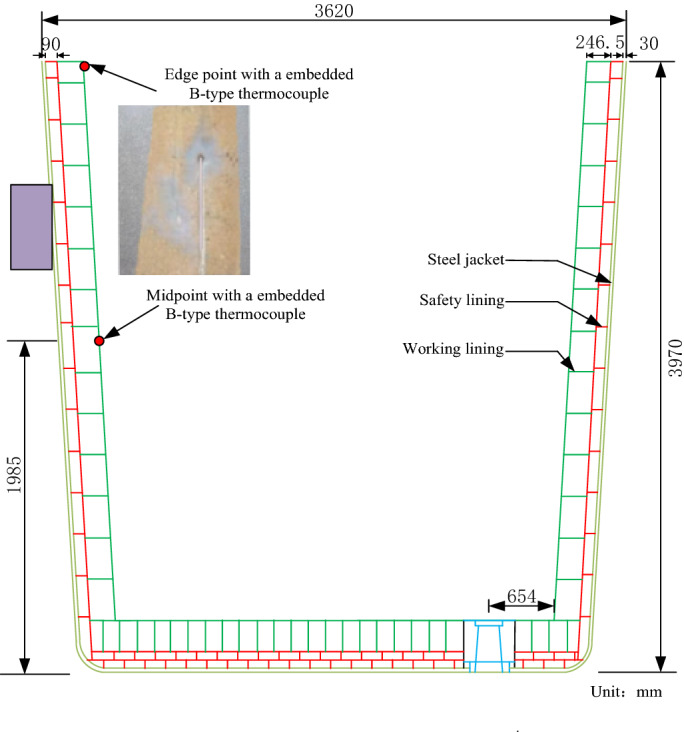
Thermocouples installation diagram.

Two ceramic tube protected B-type thermocouples are embedded on the inner wall surface of the ladle to verify the temperature field measurement. We take the edge point and the midpoint of the ladle inner wall as verification points which is shown in Fig. 8. The B-type thermocouple is the international temperature scale, and accurate temperature of the edge point and the midpoint can be acquired according to graduation table.

As a method for solving the emissivity uncertainty of the non-contact thermometry, to obtain the real temperature, the temperature probe lens should be kept clean, the lens is purged with nitrogen all the time. In order to obtain the uncertainty of the measurement method in this article during turnover process, we still select the three representative points, the bottom point, the midpoint, and the edge point along the height direction on the ladle inner wall as observation points to be measured repeatedly in 60 ms. The repeated measurement results and the standard uncertainty of the measurement of the three observation points are shown in Table 1.

**Table 1:** Repeated measurement results and the uncertainty of the measurement.

Observation points	Repeated measurement results (°C)						The uncertainty of the measurement (°C)
	*T*_1_	*T*_2_	*T*_3_	*T*_4_	*T*_5_	*T*_6_	
The bottom point	1026.6	1026.3	1026.1	1025.9	1026.5	1026.1	0.17
The midpoint	953.1	952.7	951.9	952.8	953.2	952.8	
The edge point	765.5	766.3	765.5	765.4	764.6	765.0	

In Table 1, the uncertainty of the measurement is expressed as combined sample standard deviation:

12 $$u(\overline{T}) = \frac{{\mathop \sum \nolimits_{j = 1}^{m} s(\overline{T})}}{m} = \frac{{\mathop \sum \nolimits_{j = 1}^{m} \sqrt {\frac{{\mathop \sum \nolimits_{i = 1}^{n} \left( {T_{i} - (\overline{T})} \right)^{2} }}{{n\left( {n - 1} \right)}}} }}{m}$$

where $T_{i}$ is the repeated temperature measurement result of each observation point; $\overline{T}$ is average of $T_{i}$ of each point; $n$ is the times of the measurement of each point, $m$ is the number of the observation point; $s(\overline{T})$ is measurement standard deviation.

As shown in Table 1, the uncertainty of the measuring method of this article is 0.17 °C by test statistics.

The measurement and verification results during turnover process are shown in Table 2. Because all the effective emissivity of the micro panels on inner wall of the ladle are obtained by fusion of surface spectral emissivity with temperature variation and Monte Carlo calculation model, the inner wall temperature distribution measurement achieves a good result and meets measuring needs of the filed. In the statistical results of the measuring test and verification, the maximum absolute error of the measuring temperature is 4.7 °C, the minimum error is 0.6 °C, and the average error is less than 2.8 °C.

**Table 2:** Test and contrast between the method in this article and the thermocouple.

Measurement verification point	Temperature measuring result before correction (infrared radiation pyrometer) (°C)	Temperature measuring result after correction in this article (°C)	Measuring value of the thermocouple (°C)	Absolute measurement error before correction (°C)	Absolute measurement error after correction (°C)
The edge point	824.6	856.2	854.4	29.8	1.8
	807.9	839.8	843.8	35.9	4.0
	739.1	778.9	780.1	41.0	1.2
	683.5	734.6	730.0	46.5	4.6
The midpoint	1045.8	1068.3	1069.5	23.7	1.2
	1020.5	1046.1	1043.9	23.4	2.2
	942.1	976.6	973.3	31.2	3.3
	919.3	948.3	945.3	26.0	3.0

The method in this article has solved the uncertainty of effective emissivity for non-contact thermometry, with a high accuracy close to contact thermometry, the method is convenient for installation and maintenance of the inner wall temperature measurement of the ladle in the turnover process.

## Conclusion

This article proposes a temperature field measuring method for ladle inner wall based on Monte Carlo model for effective emissivity correction. We adopt a rotating platform to drive an infrared temperature sensor and a laser sensor to scan the temperature field of the ladle inner wall at the hot repair station, then establish Monte Carlo effective emissivity calculation model of the ladle cavity to obtain the effective emissivity of each measured point on the inner wall of the ladle, therefore correct the scanning brightness temperature to the real temperature by the model. The main conclusions are as follows:

1. The principle and process of temperature distribution measurement of the ladle inner wall by scanning measurement are presented.
2. According to measurement parameters in the field, Monte Carlo calculation model for effective emissivity analysis of the ladle cavity is established. The distance between the fire baffle and the ladle is determined to be 1600 mm by effective emissivity effect analysis of the model.
3. The effective emissivity of the points on the ladle inner wall increases with the increase of the material surface emissivity of the inner wall. When the material surface emissivity exceeds 0.7, the effective emissivity becomes stable. Besides, when the material surface emissivity exceeds 0.8, the variation of the effective emissivity is less than 0.02.

As in the test and verification, contrast with the thermocouple, the maximum error of the method in this article is 4.7 °C, the minimum error is 0.6 °C, and the average error is less than 2.8 °C. It will provide reliable feedback parameters for the automatic steelmaking process with high accuracy and good application prospect.
